# The nature of fibrous dysplasia

**DOI:** 10.1186/1746-160X-5-22

**Published:** 2009-11-09

**Authors:** Liviu Feller, Neil H Wood, Razia AG Khammissa, Johan Lemmer, Erich J Raubenheimer

**Affiliations:** 1 Department of Periodontology and Oral Medicine, School of Dentistry, Faculty of Health Sciences, University of Limpopo (Medunsa Campus), Pretoria, South Africa; 2 Department of Oral Pathology, School of Dentistry, Faculty of Health Sciences, University of Limpopo (Medunsa Campus), Pretoria, South Africa

## Abstract

Fibrous dysplasia has been regarded as a developmental skeletal disorder characterized by replacement of normal bone with benign cellular fibrous connective tissue. It has now become evident that fibrous dysplasia is a genetic disease caused by somatic activating mutation of the Gsα subunit of G protein-coupled receptor resulting in upregulation of cAMP. This leads to defects in differentiation of osteoblasts with subsequent production of abnormal bone in an abundant fibrous stroma. In addition there is an increased production of IL-6 by mutated stromal fibrous dysplastic cells that induce osteoclastic bone resorption.

## Introduction

Fibrous dysplasia (FD) is a sporadic benign skeletal disorder that can affect one bone (monostotic form), or multiple bones (polyostotic form), and the latter may form part of the McCune-Albright syndrome (MAS) or of the Jaffe-Lichtenstein syndrome (JLS). JLS is characterized by polyostotic FD and café-au-lait pigmented skin lesions, while MAS has the additional features of hyperfunctional endocrinopathies manifesting as precocious puberty, hyperthyroidism or acromegaly [1,2].

Gender prevalence of FD is equal. The monostotic form is more common and affects the 20 to 30 years age group: polyostotic FD has its onset mainly in children younger than 10 years of age, the lesions grow with the child, stabilize after puberty, and most commonly involve craniofacial bones, ribs, and metaphysis or diaphysis of the proximal femur or tibia [3]. The ratio of occurrence of polyostotic to monostotic FD is 3:7 [4,5].

Signs and symptoms of FD include bone pain, pathological fractures and bone deformities [6]. Serum alkaline phosphatase (ALP) is occasionally elevated, but calcium, parathyroid hormone, 25-hydroxyvitamin D, and 1,25-dihydroxyvitamin D levels in most cases of FD are normal. Persons with extensive polyostotic FD may have hypophosphatemia, hyperphosphaturia and osteomalacia [3]. Malignant transformation is rare, and is usually precipitated by radiation therapy [7].

The craniofacial bones are affected in about 10% of cases of monostotic FD and in 50% to 100% of cases of polyostotic FD [4,8,9]. When only the cranial and facial bones are affected by FD the term craniofacial FD is used. The prevalence of the polyostotic craniofacial FD ranges from 71% to 91% and of the monostotic form, from 10% to 29% [8,9]. FD of the jaws affects the maxilla more frequently than the mandible and affects females more frequently than males [7].

**Figure 1:**
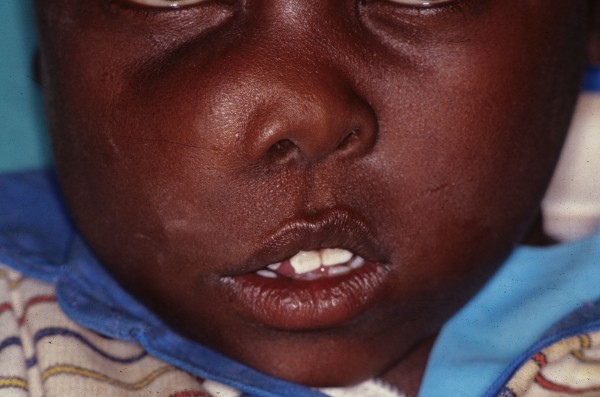
**Craniofacial fibrous dysplasia showing a diffuse swelling of the right maxillary region causing facial asymmetry**.

**Figure 2:**
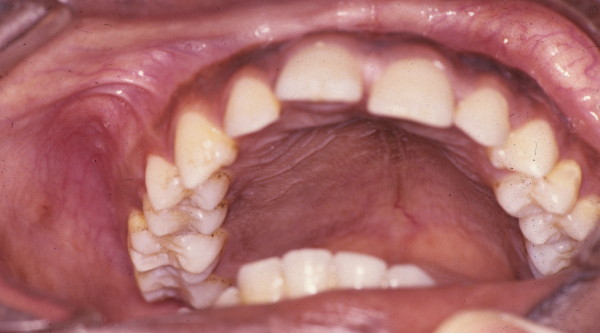
**Intraoral view of case shown in figure 1**. Note the diffuse expansion of the palate and buccal bony plate of the maxilla.

Any cranial or facial bone can be affected by FD and the clinical associated features will depend upon the bone or bones affected. Signs and symptoms can include facial pain, headache, cranial asymmetry, facial deformity, tooth displacement, and visual or auditory impairment (figures 1 and 2) [4,8].

## The aetiology of FD

FD is a genetic non-inherited condition caused by missense mutation in the gene GNAS1 on chromosome 20, that encodes the alpha subunit of the stimulatory G protein-coupled receptor, Gsα. The activating mutations occur post-zygotically, replacing the arginine residue amino acid with either a cystein or a histidine amino acid. The mutation selectively inhibits GTPase activity, resulting in constitutive stimulation of AMP-protein kinase A intracellular signal transduction pathways [2,6,10-16].

The systemic manifestations of the mutated Gsα protein-coupled receptor complex include autonomous function in bone through parathyroid hormone receptor; in skin through melanocyte-stimulating hormone receptor; in ovaries through the follicle-stimulating hormone receptor; and in the thyroid and the pituitary gland, through the thyroid and growth hormone receptors respectively [3].

FD is a somatic mosaic disorder with a broad spectrum of phenotypic heterogeneity. The extent of the disease is related to the stage at which the post-zygotic mutation in Gsα had occurred, whether during embryonic development or postnatally [13,16].

Polyostotic FD can affect bones derived from mesoderm or neural crest, and is associated with pregastrulation mutation. The same process associated with multiple-organ manifestations of Gsα mutation is referred to as McCune-Albright syndrome. The mutated pluripotential cell develops into a mutated clone of cells affecting bones in the case of FD, and affecting multiple organs together with bones in the case of McCune-Albright syndrome [6].

Monostotic FD and polyostotic FD without either craniofacial skeletal or extraskeletal organ involvement can develop from a post-gastrulation mutation; but since polyostotic FD nearly always involves craniofacial bones, it is reasonable to assume that the monostotic FD is the only form of FD that can develop post-gastrulation [6].

Severity and extent of Gsα mutation-associated diseases are not related to the stage of embryogenesis when the mutation occurred, but rather are functions of survival of mutated cells within the clone during migration, growth and differentiation, and of the ratio of mutated to normal cells at the affected anatomical site [6,13].

The postnatal manifestation of FD is not a reflection of the stage of development when the mutation occurred but indicates the time that the dynamic equilibrium between mutated and normal osteogenic cells in the mosaic fibrous dysplastic bone favoured the mutated cells. Possible factors influencing the dominance of mutated over normal cells include growth factors and hormones [6], and it is probable that there is a 'critical mass' of mutated cells necessary for the development of FD. The burden of mutated cells in FD frequently declines with age, owing to imponderable suppressive influences shifting the balance of transformed to normal cells towards predominance of normal cells, resulting in arrest of FD [6].

The cellular portion of the abnormal bone in FD is composed of a mosaic of mutated and non-mutated osteogenic cells [16]. In fibrous dysplastic bone, the increased expression of cAMP by the mutated lesional cells is associated with abnormal osteoblast differentiation and formation of defective bone [17]. Fibrous dysplastic lesions have characteristic changes in bone matrix organization, in expression of certain non-collagenous proteins of the extracellular matrix, and in mineralization; and the mutated cells within the lesion are morphologically altered [15].

## The skeletal lesions of FD

Focal lesions of FD are somatic mosaics, and the severity and extent of the bony lesions are a function of the ratio between the mutated cells and the normal osteoblasts; and of the severity of cytogenic alterations and the subsequent functional impairment of the mutated cells [3,10].

The cellular component of the bony lesions of FD comprises mesenchymal cells of osteogenic lineage. There is a variable ratio between normal osteoblasts and mutated fibroblast-like cells. The mutated cells are poorly differentiated, functionally impaired osteoblasts with an increased proliferation rate [17], and are capable of producing extracellular matrix and woven bone. However the woven bone is abnormal in organization and in composition.

The bone matrix in fibrous dysplastic lesions is deficient in osteopontin and in bone sialoprotein (BSP), compared to normal bone. BSP is a marker of osteoblastic cell differentiation and its expression is required for mineralization [2,17]. Indeed, fibrous dysplastic bone lesions demonstrate a deficit in mineralization that can be defined as localized osteomalacia. The unmineralized woven bone in long bones at sites where FD develops never matures into lamellar bone; and the local 'normal' mineralized bone adjacent to the lesion shows a relatively low mineral concentration. However, in persons with FD, the bones that are not affected by FD do not have osteomalacic changes [14,15]. In contrast to FD of long bones, in craniofacial FD the immature woven bone may undergo lamellation. These differences between the mineralization of FD of long bones and of craniofacial membranous bones, may be owing to the fact that these two embryologically distinct types of bone are under different inductive influences during development.

In addition to the osteomalacic changes, fibrous dysplastic bone shows increased osteoclastic activity, and markers of bone resorption may be elevated in some affected persons [15]. The mutated stromal cells of FD express high levels of IL-6 owing to the inherited cellular excess of cAMP. The increased levels of IL-6 stimulate osteoclastogenesis that contributes to the bone resorption at the site of FD [10]. Thus the fibrous dysplastic bone is characterized by increased bone resorption and poor mineralization.

FD and bone lesions caused by hyperparathyroidism are similar in nature, and are generated by the intracellular downstream effect of the activation of the parathyroid hormone (PTH) G protein-coupled receptor of osteogenic cells. While in hyperparathyroidism PTH receptor is over stimulated by excess PTH, in FD the same receptor is inherently active owing to the mutation in the α subunit of the G protein [12]. The end result in both FD- and in hyperparathyroidism-associated bony lesions is an increase in osteoclastogenesis resulting in bone resorption. However, while hyperparathyroidism-induced bony lesions are characterized by tunnelling bone resorption [15], there is evidence that fibrous dysplastic lesional cells are more sensitive and responsive to PTH stimulation than normal osteoblasts, but tunnelling resorption is not evident in persons with FD that do not have parathyroidism [15].

## Radiological features and microscopic features of FD

**Figure 3:**
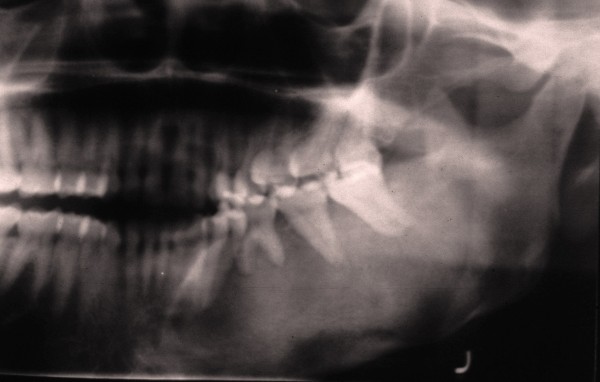
**Cropped panoramic radiograph of fibrous dysplasia of the left mandible**. Note the diffuse mottled-glass appearance and tooth displacement.

The radiological features of FD are diverse and are dependent upon the proportion of mineralized bone to fibrous tissue in the lesion [17]. Early FD of craniofacial bones is radiolucent with either ill defined or well defined borders, and may be unilocular or multilocular. As the lesions mature, the bony defects acquire a mixed radiolucent/radiopaque appearance, and established FD exhibits mottled radiopaque patterns often described as resembling ground glass, orange peel or fingerprints, with ill defined borders blending into the normal adjacent bone (figure 3) [1,9,18].

**Figure 4:**
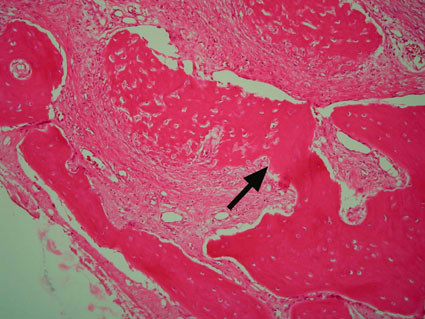
**Poorly demarcated line of fusion between FD bone (left of arrow) and residual bone (right of arrow) (H&E stain, ×100)**.

**Figure 5:**
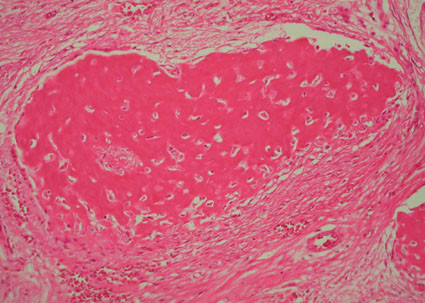
**Fibroblast-like osteoblast progenitor cells forming a woven bone deposit in a fibrous matrix**. Note the absence of osteoblastic rimming around the woven bone (H&E stain, ×250).

Microscopically, FD comprises irregular trabeculae of woven bone, blending into the surrounding normal bone (figure 4) and lying within a cellular fibrous stroma with osteoblast progenitor cells resembling fibroblasts (figure 5) [19]. These trabeculae of woven bone have been fancifully said to resemble Chinese script writing [1].

**Figure 6:**
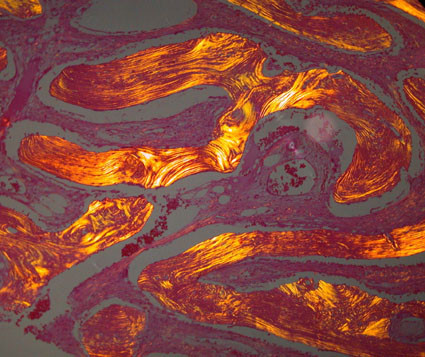
**Polarized light photomicrograph of craniofacial FD showing lamellation of Chinese character-like trabeculae (H&E stain, polarized light, ×150)**.

Early craniofacial FD is characterized by minimally mineralized deposits of woven bone with a continuum progressive lamellation of the woven bone trabeculae as FD becomes more mature (figure 6). This is in contrast to FD lesions in long bones where mature lamellar bone is not found [15].

## Treatment of FD

There is no cure for FD, and the existing guidelines for treatment are not universally accepted. Spontaneous resolution of FD does not occur [17]. Fibrous dysplastic lesions that are not symptomatic, that do not progress and that do not cause deformities or functional impairment should simply be monitored [8]. Surgical intervention is required when important structures are in danger of compression [9]. However, after surgical reduction of fibrous dysplastic lesions, particularly in younger subjects and when the lesions are more immature, is high (50%) [8,9], so a conservative surgical approach will often require more than one intervention to control the clinical signs and symptoms [8]. As an alternative treatment, when surgery is not indicated, relief of bone pain and reduction of osteoclastic activity with partial filling of osteolytic lesions can be achieved with intravenous bisphosphonate therapy [3,17,20].

## Conclusion

Fibrous dysplastic lesional cells are committed osteogenic precursor cells with impaired capacity to differentiate into normal functioning osteoblasts. The defects in osteoblast differentiation are associated with Gsα mutation of both neural crest and mesoderm-derived osteogenic cells and may thus affect any part of the osteogenic compartment.

## Competing interests

The authors declare that they have no competing interests.

## Authors' contributions

NHW, RAGK contributed to the literature review. LF, JL, NHW and EJR contributed to the conception of the article. LF, JL, NHW and RAG contributed to the manuscript preparation. EJR carried out histological analyses and drafted the histology section. Each author reviewed the paper for content and contributed to the manuscript. All authors read and approved the final manuscript.
